# Ubiquitin-specific proteases as therapeutic targets for the treatment of breast cancer

**DOI:** 10.1186/s13058-014-0461-3

**Published:** 2014-10-25

**Authors:** Anupama Pal, Nicholas J Donato

**Affiliations:** Department of Internal Medicine, Division of Hematology/Oncology, University of Michigan Comprehensive Cancer Center, 1500 E. Medical Center Drive, Ann Arbor, 48109 MI USA

## Abstract

Key mediators of signaling pathways in breast cancer involve post-translational protein modification, primarily mediated through phosphorylation and ubiquitination. While previous studies focused on phosphorylation events, more recent analysis suggests that ubiquitin plays a parallel and equally important role in several signaling and cell regulatory events in breast cancer. Availability of new tools capable of sensitive detection of gene mutations and aberrant expression of genes and proteins coupled with gene-specific knockdown and silencing protocols have provided insight into the previously unexplored ubiquitin regulatory process within these tumors. Ubiquitin-specific proteases are one class of enzymes with protein deubiquitinating activity, making up the majority of protein deubiquitinating diversity within mammalian cells. Ubiquitin-specific proteases are also emerging as potential therapeutic targets in many diseases, including cancer. In this report, we summarize the involvement of this class of enzymes in breast cancer signaling and cell regulation and illustrate the potential for additional studies to define novel targets and approaches in breast cancer therapy.

**Electronic supplementary material:**

The online version of this article (doi:10.1186/s13058-014-0461-3) contains supplementary material, which is available to authorized users.

## Introduction

Ubiquitination involves the covalent attachment of ubiquitin, a 76 amino acid protein, to numerous target proteins in a specific fashion to regulate their half-life, localization, activity and conformation [1],[2]. Given the importance of ubiquitin-mediated changes in protein function and destruction, it is not by chance that the entire process is highly regulated as small changes in this cascade lead to pathologic consequences. Disruption of the ubiquitination cycle by mutations or modified expression of specific components within the cascade has been associated with cancer, diabetes, neurologic and developmental disorders [3],[4]. Therapeutic potential thus exists for the identification of lesions within the ubiquitin cycle that can be targeted by small molecule-based approaches.

Ubiquitination is a multistep cascade catalyzed by at least three components – activation, conjugation and ligation – performed by ubiquitin-activating enzymes, ubiquitin-conjugating enzymes and ubiquitin ligases, respectively [1]. The initial research focus had been directed towards targeting the ubiquitin-activating enzymes, with activity described for small molecule inhibitors PYR-41 and PYZD-4409 [5],[6]. However, additional targets have emerged that allow more selective pathway interference. MLN4924 is a small molecule inhibitor of NEDD8-associated NAE enzyme activity that blocks neddylation-dependent cullin-RING ubiquitin ligases to induce tumor cell apoptosis [6]. MLN4926 is currently being clinically evaluated. Efforts are underway to target ubiquitin-conjugating enzymes, as exemplified by development of the ubiquitin-conjugating enzyme hCdc34 inhibitor CC0651, which is currently in preclinical development [6]. Ubiquitin ligases provide more target specificity through their selective binding to protein substrates. Several ubiquitin ligases have been linked to cancer. The classic examples are MDM2 and IAPs, among others. There has been interest in developing inhibitors against MDM2 that regulate the expression levels of tumor suppressor and proapoptotic protein p53. Nutlin-3 and JNJ-26854165 are ubiquitin ligase inhibitors that are directed against MDM2 and are currently undergoing clinical evaluation as anticancer therapy [7]. In addition, small molecule inhibitor RITA (reactivation of p53 and induction of tumor cell apoptosis), Syl-155, RO5353, RO2468 and MI-63 are other inhibitors of MDM2 that show therapeutic potential that is being further investigated [7],[8]. Seven IAP antagonists are also in phase I/phase II clinical trials [6]. However, specific efficacy of these inhibitors against one or more forms of breast cancer has not been described.

Ubiquitination is reversible, like most regulatory processes, and the enzymes that reverse protein ubiquitination are collectively known as deubiquitinases (DUBs). The mammalian genome encodes around 100 DUBs categorized into five classes, four of which are thiol proteases including ubiquitin C-terminal hydrolases (UCHs), ubiquitin-specific proteases (USPs), ovarian tumor domain DUBs and machado Joseph domain DUBs. The fifth class is represented by JAB1/MPN metalloenzyme, which functions as a zinc finger metalloprotease [9].

DUBs play a crucial role in ubiquitin processing, reversal of ubiquitin signaling and recycling of ubiquitin [10]. Through their substrate-specific deubiquitinating activity, DUBs are implicated in the regulation of critical pathways including the internalization and degradation of receptor tyrosine kinases, activity and localization of signaling intermediates, gene transcription, cell cycle progression, apoptosis, chromosomal translocation and DNA damage repair [11]-[14]. Thus it is not surprising that defective DUB activity or expression has been associated with neurological disorders and cancer.

Since USPs represent a large and diverse subset of proteins with DUB activity, much of the research has focused on assessing their function, substrates and role in specific diseases. Overall assessment of gene mutations and overexpression of USPs in different cancer types coupled with their potential for small molecule-mediated inhibition make USPs attractive as therapeutic targets, and there is growing interest in the development of USP-specific inhibitors as antiviral and anticancer agents. Since breast cancer represents a broad and diverse tumor type associated with a variety of genetic backgrounds, there is great potential for several USPs to play a role in this disease. This review examines USPs associated with breast cancer, highlights their known target actions and discusses their potential as targets in breast cancer therapy.

## Ubiquitin-specific protease-regulated signaling pathways implicated in breast cancer

An important aspect that emerges from the USPs implicated in breast cancer is that they are critical regulators of transforming growth factor beta (TGFβ) signaling, which has a well-documented role in mediating epithelial-to-mesenchymal transition (EMT), tumor progression and metastasis in breast cancer [15]. Canonical TGFβ signaling involves ligand binding to heterodimeric complexes between TGFβ receptors I and II that activate the receptor Smads, Smad2/3, which then form complexes with Smad4 coreceptor and translocate to the nucleus, activating TGFβ-dependent transcription [16]. The TGFβ signaling pathway can be regulated at multiple steps by various molecular regulatory mechanisms including ubiquitination of pathway molecules [17]. Several ubiquitin ligases such as NEDD4L and SMURF1 have been shown to attenuate TGFβ signaling by ubiquitinating Smad2 [17],[18]. Smad7 is an inhibitory Smad that regulates TGFβ signaling by recruiting ubiquitin ligases such as SMURF1/2, WWP1 and NEDD4L to the TGFβ type I receptor, leading to its ubiquitin-mediated degradation and inhibition of TGFβ signaling [17],[18]. The dichotomy of TGFβ signaling – that is, a tumor suppressor in normal cells and a tumor promoter in cancer cells – is not well understood [19]. The recent findings suggest that several DUBs may be key contributors to divergent TGFβ signaling and organization of the biologic response.

**Figure 1:**
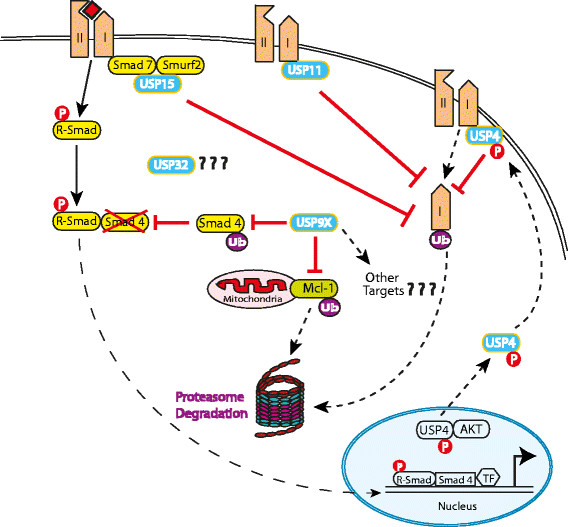
**Regulation of transforming growth factor beta signaling by ubiquitin-specific proteases in breast cancer.** Ubiquitin-specific proteases (USPs) overexpressed and implicated in breast cancer regulate transforming growth factor beta (TGFβ) signaling at different levels in the signaling cascade. USP15, USP11 and USP4 inhibit TGFβ type I receptor degradation by preventing proteasomal destruction through deubiquitination and stabilization of TGFβ type I receptor, resulting in enhancement of TGFβ signaling. USP11 directly binds to the type I receptor whereas USP15 binds the receptor through complex formation with Smad7–Smurf2. USP4 also binds directly to the type I receptor but only when phosphorylated by AKT kinase. USP4 is phosphorylated in the nucleus by AKT kinase. Phosphorylated USP4 translocates to the membrane, binds and stabilizes type I receptor. TGFβ signaling can also be regulated at the coreceptor Smad level by USP9X. Smad4 mono-ubiquitination at K519 inhibits its binding with phospho-Smad2 and thus inhibits Smad 4 and TGFβ signaling. Through its deubiquitinating activity, US9X reverses mono-ubiquitination and stabilizes Smad4, resulting in the sustained activation of TGFβ signaling. P, phosphorylation; TF, transcription factor; Ub, ubiquitin.

DUBs can regulate TGFβ signaling at the receptor, receptor-Smad or coreceptor-Smad level (Figure 1). USP11 and USP15 regulate TGFβ signaling through modulation of TGFβ receptor I levels [20],[21]. USP11 binds to Smad7, which then recruits USP11 to TGFβ receptor I where it interacts, deubiquitinates and stabilizes TGFβ receptor I to sustain Smad-mediated TGFβ signaling [20]. USP15 binds to the Smad7–SMURF2 complex, which recruits USP15 to TGFβ receptor I and stabilizes it without direct binding [21]. USP4 is reported to directly interact and deubiquitinate TGFβ receptor I to regulate TGFβ signaling [22]. Inhibition of USP11, USP4 and USP15 blocks TGFβ-mediated EMT and invasion in breast cancer [20]-[22]. Interestingly, AKT directly interacts and phosphorylates USP4, which then translocates from the nucleus to the plasma membrane where it stabilizes TGFβ receptor I through direct interaction [22]. AKT activation is associated with poor prognosis in breast cancer and inhibiting USP4 suppresses AKT-mediated breast cancer cell migration [22]. USP4 has thus been proposed as an important determinant of crosstalk between TGFβ and AKT signaling in breast cancer.

Smad4 is the central transducer of TGFβ/bone morphogenetic protein (BMP) signaling. Smad4 regulation in the cell is not completely understood but phosphorylation–dephosphorylation has been ruled out as its primary regulatory mechanism. A recent report shows that USP9X modulates Smad4 levels in cells through reduction of its mono-ubiquitination. Smad4 mono-ubiquitination at K519 prevents its binding with phospho-Smad2, inhibiting TGFβ signaling [23]. USP9X reverses Smad4 ubiquitination to reactivate the pathway and reinstate TGFβ signaling. USP9X can thus regulate TGFβ-mediated EMT and invasion through modulation of ubiquitin–Smad4 levels. USP9X can also modulate ubiquitin ligase SMURF1, a negative regulator of TGFβ/BMP signaling that controls tumor cell migration and invasion by targeting Rho family proteins [24]-[26]. SMURF1 levels are tightly regulated in the cell through multiple mechanisms, including auto-ubiquitination, which targets SMURF1 for proteasomal degradation. USP9X was identified as a novel SMURF1 interacting protein that antagonizes SMURF1 auto-ubiquitination and destabilization. Importantly, depletion of USP9X in MDAMB231 metastatic breast cancer cells, which have elevated SMURF1 expression, inhibits SMURF1-dependent breast cancer cell motility [24]. USP9X-mediated regulation of TGFβ signaling even extends to neural development [27].

Various other breast cancer-related pathways have been shown to be regulated by DUBs. USP4 regulates tumor necrosis factor alpha-induced activation of NF-κB through deubiquitination-dependent downregulation of TGFβ-activated kinase 1 (TAK1) [28]. Overexpression of USP4 inhibits TAK1-dependent NF-κB activation, whereas its knockdown is associated with enhanced tumor necrosis factor alpha-induced poly-ubiquitination of TAK1, Iκ phosphorylation and NF-κB-dependent gene expression. USP11 has been shown to regulate tumor necrosis factor alpha-mediated NF-κB activation through modulation of IκBα stability [29]. USP15 reverses the ubiquitinating activity of CSN on IκBα, thus negatively regulating NF-κB signaling [30].

## Ubiquitin-specific proteases overexpressed in breast cancer

A list of USPs overexpressed in breast cancer is provided in Table 1.

**Table 1:** **Ubiquitin-specific proteases overexpressed in breast cancer**

Deubiquitinase	Upregulated/downregulated	Breast cancer context	Implicated signaling	References
USP9X	Upregulated	Human ductal carcinomas	TGFβ signaling	[23],[42],[43]
	Upregulated	Human breast cancer tissue as compared with the adjacent normal tissue		
USP15	Upregulated	Tissue microarray of 23 breast tumors	TGFβ signaling	[21],[32]
USP32	Upregulated	Copy number alterations in ER + human breast tumors,		
		50% (nine of 18) of breast cancer cell lines and 22% (nine of 41) of primary breast tumors compared with mammary epithelial cells.		[34],[35]
		*USP32–CCDC49* expressed fusion gene in ER+, tamoxifen-resistant breast cancer cell line ZR-75-30		
USP9y, USP10, USP25	Upregulated	Human breast cancer tissue as compared with the adjacent normal tissue		[42]
USP4	Upregulated	KD inhibits EMT, cell migration, invasion and metastasis in human breast cancer cells *in vitro* and *in vivo* in zebrafish xenograft metastasis model	TGFβ signaling	[22]
USP11	Upregulated	KD inhibits TGFβ-induced EMT in normal mouse mammary epithelial cells (NMuMG)	TGFβ signaling	[20]

ER+, estrogen receptor-positive; EMT, epithelial-to-mesenchymal transition; KD, knockdown; TGFβ, transforming growth factor beta; USP, ubiquitin-specific protease.

### Ubiquitin-specific protease 15

The *USP15* gene is found amplified in around 2% of human breast tumors as well as other tumors, including ovarian and glioblastoma tumors [21]. Recent data implicate USP15 as an important regulator of cell cycle progression [31]. In glioblastomas, USP15 is reported to bind to the SMAD7–SMURF2 complex and stabilizes the TGFβ type I receptor through deubiquitination to enhance TGFβ signaling [21]. USP15 is required for TGFβ-mediated signaling and cell motility in the MDAMB231 metastatic breast cancer cell line [32]. Following TGFβ ligand stimulation in breast cancer cells, SMURF2 is degraded in a TRAF4-dependent manner that facilitates recruitment of USP15 to TGFβ receptor I to sustain TGFβ signaling. The TGFβ receptor–TRAF4 interaction triggers Lys63-linked TRAF4 poly-ubiquitination and subsequent activation of TAK1. TRAF4 is thus required for efficient TGFβ-induced migration, EMT and breast cancer metastasis in a USP15-regulated fashion [33]. Most recently, USP15 was shown to stabilize MDM2 with impact on both p53 and the T-cell transcription factor NFATc. USP15 targeting may thus evoke both direct and indirect antitumor responses [7].

### Ubiquitin-specific protease 32

The *USP32* gene localizes on chromosome 17q23, which is commonly amplified in breast cancer. The interest in USP32 as a therapeutic target was generated following the study of Zhang and colleagues, which identified *USP32* as the prognostic gene with copy number alterations in estrogen receptor-positive human breast tumors [34]. High USP32 transcript levels were also reported in 50% (nine of 18) of breast cancer cell lines and 22% (nine of 41) of primary breast tumors compared with mammary epithelial cells [35]. Further, stable silencing of USP32 expression reduced proliferation and migration in the estrogen receptor-positive breast tumorigenic cell line, MCF7. Since *USP32* mutations were not detected in this cell line, it appears that the increased USP32 levels are due to amplification of the wild-type *USP32* gene [35]. The role of USP32 as a causal agent in breast cancer is further emphasized by a recent study that identified *USP32–CCDC49* as one of the nine expressed fusion genes by structural analysis of the genome of ZR-75-30, an estrogen receptor-positive breast cancer cell line that has been used as a model system to study estrogen receptor-positive breast cancers that are insensitive to tamoxifen [36].

### Ubiquitin-specific protease 4

USP4 has been reported to have higher expression levels in metastatic breast carcinomas as compared with the normal breast samples [22]. USP4 stimulates the TGFβ-mediated EMT, invasion and metastasis. Depletion of USP4 in highly metastatic MDA-MB-231 breast cancer cells leads to the depletion of EMT markers and TGFβ-induced migration *in vitro*, and *in vivo* in a zebrafish xenograft invasion metastasis model [22].

### Ubiquitin-specific protease 11

USP11 was identified as a BRCA2-interacting protein [37]. *BRCA2* is an important tumor suppressor gene that functions in double-strand DNA damage repair by homologous recombination [38]. Individuals with *BRCA2* mutations are predisposed to breast cancer [39]. USP11 has been shown to be a contributing factor in the DNA damage repair functions in the BRCA2 pathway through direct interaction, but independent of BRCA2 deubiquitination [37]. USP11-silenced cells show DNA damage repair activation even in the absence of any DNA damage and are hypersensitive to genotoxic stress-inducing agents including poly(ADP-ribose) polymerase inhibition and ionizing radiation [40]. These cells also show defective homologous recombination with misregulation of recruitment of double-strand break repair proteins including RAD51 and 53BP1 [37]. Underscoring the pro-survival role of USP11 in breast cancer, USP11 knockdown in normal mouse mammary epithelial cells (NMuMG) inhibited TGFβ-induced EMT [20]. A recent study showed that breast cancer patients with high-level USP11 expression have higher rates of recurrence and poor survival outcomes when compared with low-level USP11 expressers [41].

## Other ubiquitin-specific proteases implicated in breast cancer: USP9X, USP10 and USP25

An analysis of differential *USP* gene expression in breast cancer found greater than threefold overexpression of USP9X, USP10 and USP25 in human breast cancer tissue as compared with adjacent normal tissue [42]. Of these, *USP9X* is a well-characterized gene that has been shown to be a critical mediator of cell survival. Increased USP9X expression is reported in human follicular lymphoma and correlates with poor prognosis in multiple myeloma [43]. Some cancers, including primary breast cancer, demonstrate an association between USP9X and Mcl-1, a pro-survival BCL2 family member that is essential for stem and progenitor cell survival [43]. High Mcl-1 levels contribute to chemo-resistance and radio-resistance, contribute to disease relapse and correlate with poor prognosis in breast cancer and other cancers [44]-[46]. USP9X stabilizes Mcl-1 by preventing its recognition by the proteasome, and USP9X knockdown sensitizes cells to killing by apoptosis-inducing drugs like the BH3 mimetic ABT-737 and by radiation [43],[47],[48]. Radiation and DNA-damaging agents are also more cytotoxic in colon cancer cells with full genetic disruption of the *USP9X* gene, but these effects are independent of Mcl-1 stability [49]. Similarly, USP9X expression is required to maintain growth of glioblastomas and medulloblastomas, but USP9X-dependent growth does not appear to involve Mcl-1 stabilization [50]. The role of USP9X in breast cancer and its spectrum of breast cancer targets have not been studied [23],[42],[43]. This investigation may be particularly important because several mediators of breast cancer signaling and tumorigenicity, in addition to Mcl-1, are regulated by USP9X.

USP10 was recently reported to be a novel regulator of cellular p53 [51]. USP10 deubiquitinates p53 and reverses MDM2-mediated nuclear transport and degradation of p53. Yuan and colleagues found diminished levels of USP10 in most renal cell carcinomas and the cell lines established from them, which lack *p53* mutations. However, a small subset of renal cell carcinomas with *p53* mutations contain elevated levels of USP10 that were found to exert an oncogenic activity in cultured tumor cells. USP10 functions to stabilize both wild-type and mutant p53, and in that context can act as a tumor suppressor or an oncoprotein [51]. This function should be considered in assigning a role for this DUB in breast cancer because many breast tumors, particularly those with triple-negative status, have mutations in *p53*. USP10 also antagonizes c-myc transcription through deubiquitination of SIRT6, a histone deacetylase, and NF-κB signaling through deubiquitination of NEMO [52].

The function of USP25 in normal physiology and cancer is unknown, although recent studies suggest this protease plays a regulatory role in inflammation and innate immunity [53]. As with USP10, additional studies in breast cancer are needed for USP25.

## Small molecule inhibitor-mediated targeting of ubiquitin-specific proteases

Several partial and specific inhibitors have been developed against USPs. HBX 41,108 is a partially-selective USP inhibitor that stabilizes p53 in HEK293 cells and induces caspase 3 and PARP cleavage in both *p53*^*+/+*^ and *p53*^*−/−*^ HCT-116 cells [54]. HBX 41,108 specificity is limited because it inhibits USP5, USP8, UCH-L3 and caspase 3 in addition to USP7, a DUB that was initially identified as the sole target of HBX 41,108 [54]. P5091 is a novel USP7-specific small molecule inhibitor that induces apoptosis in multiple myeloma cells resistant to conventional and bortezomib therapies through stabilization of p53. P5091 is an active antitumor agent in various tumor models [55]. b-AP15, also called VLX1500, is a proteasome inhibitor that inhibits the activity of the 19S regulatory particle-associated DUBs, such as UCHL5, a ubiquitin C terminal hydrolase, and USP14 [55]. b-AP15 is shown to effectively inhibit tumor progression in multiple solid tumor mouse models and dissemination in acute myelogenous leukemia *in vivo* models [56]. Pimozide is a USP1 inhibitor that inhibits leukemic cell growth through degradation of ID1 proteins [57]. ML323 is a highly potent USP1 inhibitor with excellent selectivity against several DUBs that potentiates cisplatin cytotoxicity in nonsmall-cell lung cancer and osteosarcoma cells [58].

WP1130 is a partially selective DUB inhibitor that has been developed in our laboratory. WP1130 induces apoptosis in cells through rapid accumulation of poly-ubiquitinated proteins. The known targets of WP1130 include USP5, USP9X and USP14, among others. We have shown previously that USP9X inhibition by WP1130 reduces MCL-1 levels, promotes apoptosis and increases tumor cell sensitivity to chemotherapy [59]. WP1130 was also shown recently to inhibit the growth of ERG-positive tumors *in vitro* and in mouse xenograft models of prostate cancer through inhibition of USP9X [5]. We have recently identified and developed a compound with improved specificity towards USP9X and antitumor activity in mouse models of myeloma, lymphoma and melanoma (unpublished data, Potu H and Donato NJ, 2014).

We have found that lentivirus-mediated USP9X knockdown in tumorigenic human breast cancer cell lines inhibits their growth. More importantly, USP9X knockdown in all triple-negative breast cancer cell lines tested causes apoptosis induction (unpublished data, Pal A and Donato NJ, 2014). In light of these findings, WP1130 and its derivatives could be significant as therapeutic modalities in the treatment of breast cancer.

## Conclusion

USPs are a highly specialized and important class of DUBs with emerging therapeutic potential in breast cancer. Recent description of selective small molecule inhibitors for a small number of DUBs provides feasibility for targeting USPs for therapeutic purposes [60],[61]. Targeting USPs as breast cancer treatment is especially promising due to the recent expansion of the role of several USPs in the regulation of various cancer-related pathways such as TGFβ signaling at multiple levels within the pathway. Thus, it is anticipated that therapeutic control of that pathway could be achieved with USP inhibitors, which may add to future treatment options for breast cancer patients.

## Authors’ original submitted files for images

Below are the links to the authors’ original submitted files for images. Authors’ original file for figure 1

## Abbreviations

DUB: Deubiquitinase
EMT: Epithelial-to-mesenchymal transition
NF: Nuclear factor
TAK1: Transforming growth factor beta activated kinase 1
TGFβ: Transforming growth factor beta
UCH: Ubiquitin C-terminal hydrolase
USP: Ubiquitin-specific protease
