# Supplementary figures and images for: Ubiquitin-specific proteases as therapeutic targets for the treatment of breast cancer
Source: Breast Cancer Res. 2014 Oct 25;16:461. doi: 10.1186/s13058-014-0461-3 (PMC4384352; doi:10.1186/s13058-014-0461-3)

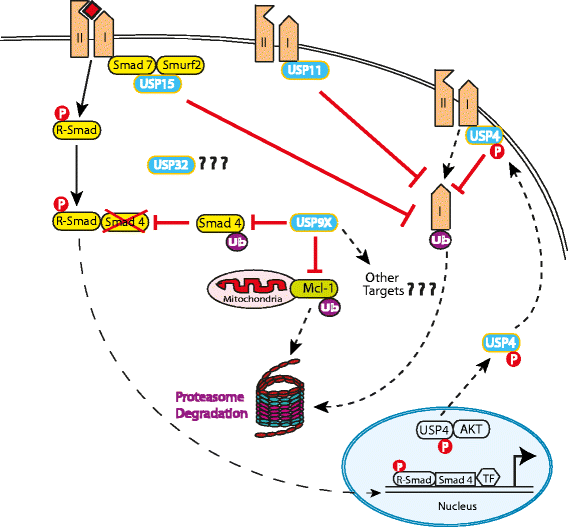

Supplement: Supplementary file 1 — Authors’ original file for figure 1 [file 13058_2014_461_MOESM1_ESM.gif]
